# Growth competence develops independently of cell division: uncoupling dormancy and cell cycle in grapevine buds

**DOI:** 10.1093/aob/mcag218

**Published:** 2026-07-18

**Authors:** Dina Hermawaty, Peta L Clode, John A Considine, Santiago Signorelli, Michael J Considine

**Affiliations:** The UWA Institute of Agriculture, The University of Western Australia, Perth, WA 6009, Australia; Department of Biotechnology, Indonesia International Institute for Life Sciences, Jakarta 13210, Indonesia; Centre for Microscopy, Characterisation & Analysis, The University of Western Australia, Crawley, WA 6009, Australia; The UWA Institute of Agriculture, The University of Western Australia, Perth, WA 6009, Australia; Departamento de Biología Vegetal, Universidad de la República, Montevideo 12900, Uruguay; The UWA Institute of Agriculture, The University of Western Australia, Perth, WA 6009, Australia; The School of Molecular Sciences, The University of Western Australia, Perth, WA 6009, Australia; The Department of Primary Industries and Regional Development, The Government of Western Australia, Perth, WA 6000, Australia

**Keywords:** Cell cycle, dormant bud, grapevine, mitotic index, ultrastructure

## Abstract

**Background and Aim:**

Winter chilling has long been considered the primary driver of bud dormancy release in woody perennials. However, the cellular mechanisms underlying dormancy transitions remain poorly understood. This study aimed to investigate the relationship between dormancy depth, cell cycle activity and cellular ultrastructure in grapevine buds across the dormancy cycle.

**Methods:**

Bud dormancy progression in single-node explants of *Vitis vinifera* cv. ‘Cabernet Sauvignon’ was monitored from early autumn dormancy to the end of winter. Dormancy depth was quantified as the time to 50 % bud burst under forcing conditions. Cell cycle status was assessed using quantitative flow cytometry, while the cellular ultrastructure of the shoot apical meristem was examined by transmission electron microscopy.

**Key Results:**

Dormancy depth declined dramatically from over 280 d in early autumn to ∼50 d in late autumn, despite negligible chilling exposure during this period. Following chilling exposure in winter, the depth of dormancy declined to ∼20 d. Strikingly, flow cytometry revealed that the majority of cells remained arrested in the G1 phase of mitosis throughout this period, regardless of dormancy depth, supported by ultrastructural analysis of the shoot apical meristem. Starch grains were abundant in both deeply dormant pre-winter buds (no chilling) and winter buds (with chilling), indicating that starch dynamics may be regulated independently of both dormancy status and chilling exposure. These findings demonstrate temporal uncoupling of dormancy release from cell cycle reactivation, challenging assumptions that dormancy release is directly linked to both chilling and cell cycle activation.

**Conclusions:**

Our results establish that in grapevine the transition to growth competence occurs independently of detectable cell division and can precede significant winter chilling accumulation. This work provides new insights into the cellular basis of dormancy regulation in woody perennials and establishes a methodological framework for dissecting this relationship in other species.

## Graphical Abstract

**Graphical Abstract:**
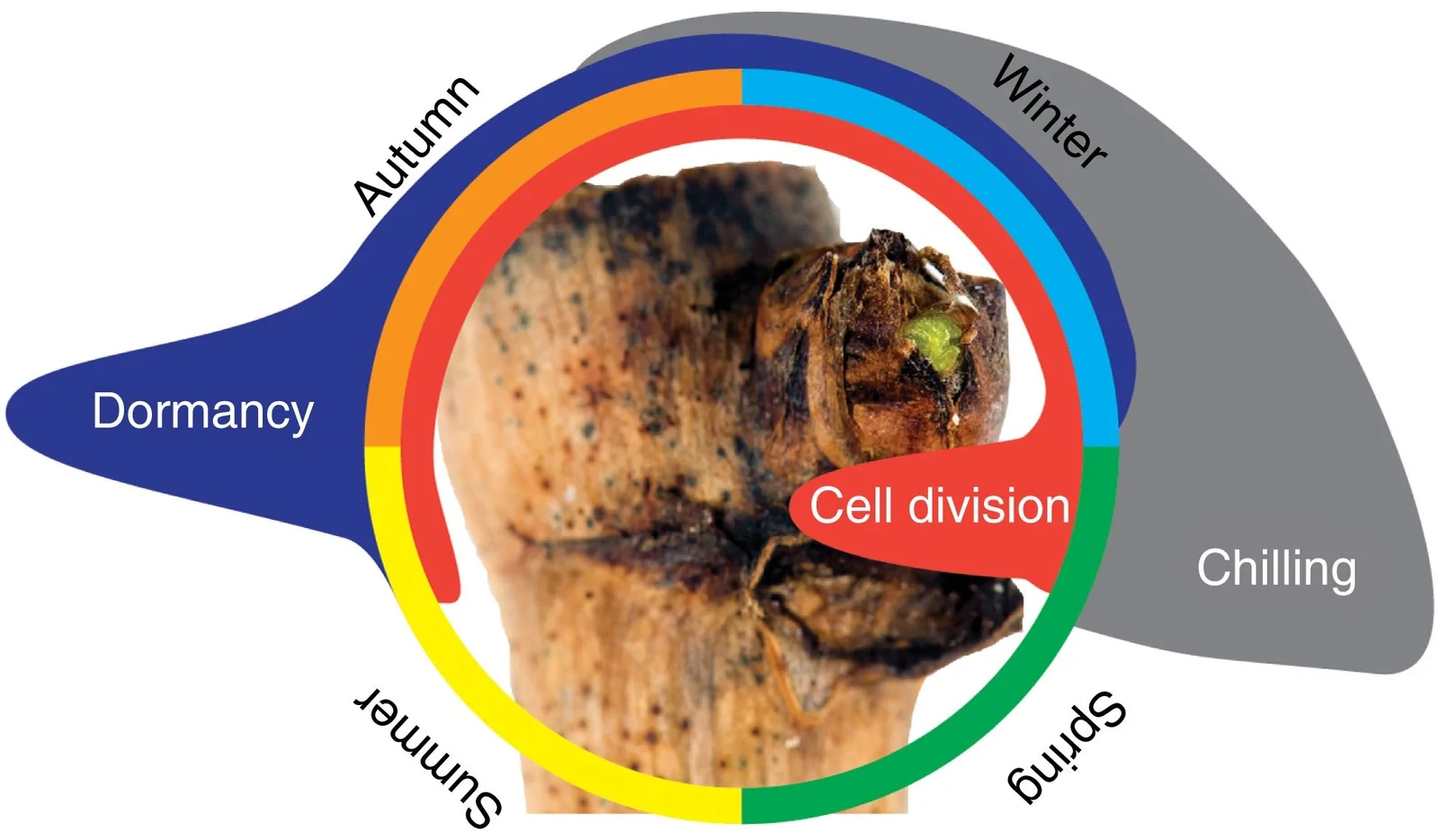

## INTRODUCTION

Bud dormancy in woody perennials has long been viewed through the paradigm of chilling-induced release, where winter cold exposure is considered essential for breaking dormancy (Saure, 1985; Heide and Prestrud, 2005). However, studies in grapevine suggest a more complex regulation, with significant physiological changes occurring before winter chilling (Pouget, 1963; Nigond, 1967; Velappan *et al.*, 2022; Signorelli *et al.*, 2026). Most notably, the depth of dormancy can decline substantially prior to winter, posing a challenge to the chill-dependent models of dormancy regulation.

Recent comprehensive analysis of seasonal changes in grapevine buds has revealed distinct physiological phases throughout the annual cycle (Signorelli *et al.*, 2026). Buds transition from an early summer quiescence, characterized by high metabolic activity, through a late summer dormant phase marked by peak dormancy depth and suppressed respiration, to a late winter quiescent phase featuring metabolic reactivation and sugar mobilization. Importantly, cell cycle analysis across this annual progression showed that cells remain predominantly in G1 phase from summer through mid-winter. Cell cycle reactivation, evidenced by a doubling of the G2/G1 ratio, occurs only in early spring (August–September in the southern hemisphere), as buds approach the natural timing of bud burst (Signorelli *et al.*, 2026). This late-season shift towards proliferation coincides with increased respiration and sugar–phosphate accumulation, suggesting coordinated metabolic and cell cycle activation preceding bud burst.

Nevertheless, the cellular and ultrastructural changes occurring during the critical earlier period, when dormancy depth declines dramatically from late summer through mid-winter, remain poorly characterized. Several studies have documented changes in the expression of cell cycle genes and transcription factors during grapevine dormancy transitions (Díaz-Riquelme *et al.*, 2012; Vergara *et al.*, 2016, 2017; Dong *et al.*, 2025), yet physiological data on actual cell division patterns (e.g. mitotic index/flow cytometry-based cell cycle profiling) and linked subcellular organization across the transitions of dormancy and quiescence remain limited (Velappan *et al.*, 2017).

Studies in seeds and embryos have established that cells typically arrest in G1 phase during dormancy (Velappan *et al.*, 2017). This arrest is well understood in seeds, where extreme desiccation restricts metabolic activity (Bewley, 1997; El-Maarouf-Bouteau, 2022). By contrast, buds maintain a substantially more hydrated state than seeds (∼40 vs ∼10 %), and grapevine buds remain connected via the apoplast to the parent plant throughout dormancy and quiescence (Signorelli *et al.*, 2020, 2026), raising questions about whether the cell cycle is similarly restricted during dormancy in latent buds. In addition, there is limited knowledge of the ultrastructural changes accompanying dormancy transitions in grapevine. In Norway spruce, dormancy onset coincides with the formation of thick-walled cells that restrict water transport (Lee *et al.*, 2017), yet similar data in grapevine are absent.

The shoot apical meristem (SAM) generates the tissue mass that emerges during bud burst through cell division at the shoot primordia (Sussex, 1989; Kean-Galeno *et al.*, 2024). The SAM’s central zone maintains pluripotent cells that supply the peripheral zones where differentiation occurs (Lenhard and Laux, 1999; Demesa-Arevalo *et al.*, 2024). This organization creates distinct cytological zones with different cell cycle activities and ultrastructural features (Meyerowitz, 1997; Bowman and Eshed, 2000; Xue *et al.*, 2020). However, how these zones respond during the transition from deep dormancy to winter quiescence, when dormancy depth declines substantially yet chilling remains minimal, is poorly understood.

In this study, we examined cell cycle status and cellular ultrastructure of grapevine buds from early autumn (March) through mid-winter (August) in the southern hemisphere, spanning the period when dormancy depth declines from over 200 d to less than 30 d despite minimal chilling accumulation. By combining quantitative flow cytometric analysis of nuclear DNA content with detailed transmission electron microscopy (TEM) of SAM ultrastructure, we reveal that cells remain predominantly arrested in G1 phase throughout this period regardless of dormancy depth, and that starch accumulation occurs in both deeply dormant pre-winter buds and chilling-exposed winter buds. These findings demonstrate that the physiological capacity for growth resumption develops independently of detectable cell cycle reactivation during the dormancy-to-quiescence transition, and that starch dynamics are regulated independently of both dormancy status and chilling exposure. This work provides new insights into the cellular basis of dormancy regulation in woody perennials and establishes that the mechanisms governing dormancy depth are fundamentally distinct from those controlling cell cycle activation.

## MATERIALS AND METHODS

Unless otherwise stated, all chemicals were obtained from Sigma–Aldrich, NSW, Australia.

### Plant material

Buds of grapevine (*Vitis vinifera* cv. ‘Cabernet Sauvignon’) were harvested from vines within a commercial cane-pruned vineyard in the temperate/Mediterranean region of Margaret River (Csb – temperate, dry summer, warm summer; Peel *et al.*, 2007), Western Australia (34°S, 115°E) on six dates, from early autumn to mid-winter of 2017 and 2018; i.e. 5 March 2017, 16 May 2017, 8 August 2017, 5 March 2018, 13 May 2018 and 4 August 2018. These dates were chosen as preliminary data showed a 6-fold range in the depth of dormancy, measured as the time for 50 % of buds to burst (BB_50_) to the stage when a green leaf tip becomes visible or EL4 of the modified Eichorn–Lorenz scale (Coombe, 1995) under forcing conditions (20 °C with a 12-h photoperiod). Canes were cut from the vines within 2 h from sunrise and trimmed to five nodes from the fourth to the eighth node (the first clear node should be ≥10 mm from the base of the branch). The canes were wrapped in damp newsprint and immediately transported to the laboratory in an insulated box and stored at 22 °C for up to 24 h. The canes were cut into short single-node cuttings of 50–70 mm length, retaining 5–10 mm above the node, as previously described in Meitha *et al.* (2015). Treatment with hydrogen cyanamide (cyanamide, H_2_CN_2_) was done by submerging the node in 1.25 % (w/v) cyanamide for 30 s as a control for the viability of buds. Control buds were treated in the same manner with water. The cuttings were then stored in the dark for 24 h before processing and subsequent analysis.

### Chilling accumulation

Hourly air temperature data were obtained from the Wilyabrup automatic weather station (station code MR001; 115.064°E, 33.792°S, https://weather.agric.wa.gov.au/station/MR001) for the period 1 January 2017 to 31 December 2018. Two complementary metrics were calculated for each season. We summed chilling hours (CH; hourly observations for which mean air temperature was strictly below 7.2 °C) across the season (Weinberger, 1950). The number of daily positive chill units (DPCU) was calculated following Linsley-Noakes and Allan (1994). Data were analysed in RStudio (v. 2025.09.2+418; R Core Team, 2024) using the dplyr and tidyr packages (Wickham *et al.*, 2023; Wickham and Vaughan, 2024). Cumulative accumulation of both metrics was plotted against calendar date using ggplot2 (Wickham, 2016).

### Depth of dormancy assessment

Fifty single-node cuttings per treatment were used to measure the depth of dormancy at each harvest time. The single-node cuttings were planted in potting mix (fine composted pine bark, coco peat and brown river sand in the ratio of 2.5:1:1.5 (w/w), pH ∼6.0), in well-drained seedling trays. Cuttings were grown in a controlled environment room at 20 °C with a 12-h photoperiod (450 µmol m^2^ s^1^ photosynthetically active radiation from Eye Hortilux 1000-watt lamps; Hortilux, OH, USA). Trays were watered every 2 d to 80 % field capacity (Signorelli *et al.*, 2022). The bud burst (EL4) date was recorded every day for up to 300 d. Non-burst buds were dissected at the end of the assessment period to observe the viability of buds.

### Flow cytometry

Nucleus preparation, flow cytometry (FCM) data acquisition and data analysis were performed as described by Hermawaty *et al.* (2022). FCM using buds was performed in three biological replicates in which ten buds were pooled in each biological replicate. Two canes from two independent vines with five buds each were used to make a pool of ten primary buds for one replicate. The single bud is the biological unit of interest, but considering that nuclei represent a minority population within the nuclear suspension, in this experiment it was necessary to pool the buds to obtain a sufficient number of nuclei for FCM analysis.

Primary buds were manually dissected under a dissecting microscope, carefully removing the secondary and tertiary buds and woolly hairs as much as possible. A total of ten primary buds (±100 mg) per replicate were collected into a plastic Petri dish (diameter 100 mm) and placed on ice. Nuclei were extracted by individually chopping samples using a sharp razor blade in 3 mL cold nuclear isolation buffer (NIB; 10 mm MgSO_4_.7H_2_O, 50 mm KCl, 5 mm HEPES and 1 mg mL^−1^ dithiothreitol (DTT), 0.5 % Triton X-100 and 3 % PVP-40 (w/v)) followed by an hour of incubation on ice. After incubation, samples were filtered through a 100- and 41-µm nylon mesh filter (Millipore, Germany), sequentially, and centrifuged at 450 *g* for 8 min at 4 °C. The nuclear pellet was resuspended in 1 mL fresh NIB followed by the addition of 50 µg mL^−1^ RNase A (Thermo Scientific, Australia, EN0531) to remove RNA from the nuclear suspension. After RNase treatment, a final concentration of 20 µg mL^−1^ propidium iodide (PI) was added to stain the nuclei. The nuclear suspension without the addition of PI served as the negative control. A mixed nuclear suspension sample was prepared for instrument setting by co-chopping 50 mg of buds (prepared by dissection as described above) with 25 mg of tomato leaves.

The nuclear DNA content was measured using a BD FACSCanto™ II (BD Biosciences, San Jose, CA) flow cytometer equipped with an air-cooled 488-nm solid state 20 mW laser. Propidium iodide was excited using blue laser light (488 nm) and the fluorescent signal was collected through a 556-dichroic long-pass filter and a 585/42-nm band-pass filter. The analysis was run in low-speed mode to collect up to 15 000 single PI-positive nuclei per biological replicate. The percentage of cells in each phase was estimated using the Watson pragmatic algorithm in FlowJo V10 (Tree Star Inc., Ashland, OR, USA) software. The quality of the nucleus preparation was evaluated using the coefficient of variance (CV), background debris factor (DF) and percentage of intact nuclei as previously described (Hermawaty *et al.*, 2022).

### Ultrastructure observation of the primary buds

Using TEM, sample preparation and observation were performed at the Centre for Microscopy, Characterisation and Analysis (CMCA), University of Western Australia. A minimum of three buds from independent canes were processed for TEM at each time point for each treatment condition. For each bud, median longitudinal sections through the SAM were examined, with representative images selected. Following the treatment with cyanamide or water, as described in the Plant material section, primary buds were manually dissected as previously described then immersed in 1.6 % formaldehyde and 2.5 % glutaraldehyde in phosphate-buffered saline (PBS) pH 7.4 solution. Infiltration of the fixative solution was done by applying a 2500 mmHg vacuum cycle for 15 min (5 min vacuum/release/mixing) at room temperature using a PELCO^®^ vacuum system fitted in the PELCO Biowave^®^ microwave processor followed by incubation at room temperature for 24 h (without vacuum). Fixed buds were kept at 4 °C until required. Prior to post-fixation with osmium tetroxide, fixed buds were further dissected for removal of secondary and tertiary buds, bud scales and trichomes (Supplementary Data Fig. S1). Post-fixation, dehydration, resin infiltration and sample embedding were performed as described by Clode (2015; Supplementary Data File S1). The microscopic study was performed on median longitudinal sections (transverse relative to the cane; Supplementary Data Fig. S1) of the zone of the SAM of the buds. Semi-thin sections at 1 µm were prepared using the resin-embedded buds using a glass knife on an EM UC6 ultramicrotome (Leica Microsystems GmbH, Wetzlar, Germany). Sections were mounted on glass slides, stained with toluidine blue (pH 9; 0.5 % toluidine blue in 1 % borax) and observed under a bright-field microscope for general morphology. Bright-field images were acquired using an Axioskop optical microscope (Carl Zeiss Spectroscopy GmbH, Jena, Germany) fitted with an Axiocam digital camera. Ultrathin sections at 100 nm were prepared using a 45° Histo Diamond Knife (Diatome, PA, USA) on an EM UC6 ultramicrotome (Leica Microsystems GmbH). Sections were mounted into unfilmed 75-mesh copper grids (ProSciTech, Queensland, Australia) and imaged unstained at 120 kV using a JEM-2100 TEM (JEOL, Tokyo, Japan). Images were acquired on an ORIUS SC1000 digital camera (Gatan, Pleasanton, CA, USA).

### Data analysis

Statistical analyses were performed using three biological replicates (*n* = 3) per condition. Data are presented as mean ± standard deviation. Differences among groups were evaluated using one-way analysis of variance (ANOVA) followed by Tukey’s multiple comparison test, with <0.05 considered significant.

## RESULTS

To investigate the relationship between dormancy state and cellular activity in grapevine buds, we analysed three key time points in the annual growth cycle – March (early autumn), May (late autumn) and August (winter) – which represent distinct physiological states in Mediterranean climates. Previous studies suggest ‘Cabernet Sauvignon’ requires ∼100 CH for dormancy induction and a further 3–400 CH units for dormancy release (Anzanello *et al.*, 2018, 2021). In our study, zero chilling exposure had accumulated prior to March or May sample dates, and <400 DPCU or 10 CH units according to the base 7.2 °C model (Supplementary Data Fig. S2). Despite this, Table 1 shows that the greatest depth of dormancy was observed in the March samples, with water-treated buds achieving only 24 % bud burst over the 300-d observation period in 2017. Cyanamide was used as a positive control, showing the high degree of viability, reaching 50 % bud burst (BB_50_) within 49 d, and 90 % of buds burst within 300 d. In the May samples, water-treated buds reached BB_50_ within 46 d and cyanamide-treated buds within 18 d. The shortest duration to achieve BB_50_ was observed in the water- and cyanamide-treated buds collected in August: 21 and 24 d, respectively. The dormancy analysis in 2018 was consistent with that in 2017.

**Table 1.** Comparison of depth of dormancy from early autumn (March) to late winter (August) in grapevine buds in single-node forcing conditions.

Harvest date	BB_50_ (d)		Bud burst (%)	
	Water treatment	H_2_CN_2_ treatment	Water treatment	H_2_CN_2­_treatment
5 March 2017	[365]	49	24	90
16 May 2017	46	18	84	100
8 August 2017	21	24	96	74
5 March 2018	[286]	27	32	86
13 May 2018	57	20	96	96
4 August 2018	24	19	100	100

Buds were treated with or without (water treatment) hydrogen cyanamide (H_2_CN_2_) and the number of days until 50 % bud burst (BB_50_) and bud burst percentage were recorded.

Numbers in square brackets for untreated buds sampled in March indicate the duration of assessment; these buds did not reach 50 % bud burst, and after 365 or 286 d the remaining buds were necrotic.

### Cell cycle profile of dormant buds

Having established that dormancy depth varies significantly across the growing season, with a dramatic decline from March to May before a more gradual decline to August, we next investigated whether these physiological changes were reflected in cell cycle activity. The proportion of cells in each cell cycle phase was estimated from the univariate PI DNA histogram (Supplementary Data Fig. S3) using the Watson pragmatic algorithm (Watson *et al.*, 1987). Results show that the majority of cells were in the G1 phase of the cell cycle in both years, although there were marginal differences in magnitude between years (Table 2). There was no significant effect of cyanamide on the mitotic index within the 24 h of treatment in either 2017 or 2018. The mitotic index (proportion of G2/G1 cells) declined significantly from March to May and to August in 2017. While the trend was consistent in 2018, the differences were not significant. The CV value for the cytometric data and debris level in the nuclei suspension ranged from 10 to 14 % and 59 to 79 %, respectively.

**Table 2.** Proportion of cells in each cell cycle phase in grapevine buds collected in 2017 and 2018.

Harvest date	Treatment	G1 (%)	S (%)	G2 (%)	Mitotic index (G2/G1)	CV (%)	Debris factor
5 March 2017	Water	43.30 ± 6.55a	14.31 ± 5.37ab	39.33 ± 1.12c	0.92 ± 0.11c	12.87 ± 1.53ab	0.64 ± 0.01a
5 March 2017	H_2_CN_2_	38.63 ± 5.27a	23.47 ± 7.26b	31.80 ± 4.20b	0.82 ± 0.04c	10.25 ± 2.12a	0.59 ± 0.03a
16 May 2017	Water	61.74 ± 5.51b	9.20 ± 5.20a	29.08 ± 1.20b	0.47 ± 0.05b	14.24 ± 1.65b	0.61 ± 0.02a
8 August 2017	Water	65.27 ± 3.45b	14.21 ± 4.42ab	20.30 ± 2.29a	0.31 ± 0.03a	11.93 ± 1.80ab	0.63 ± 0.02a
5 March 2018	Water	62.37 ± 3.40a	14.60 ± 2.00a	22.33 ± 5.25a	0.36 ± 0.11a	11.10 ± 0.62a	0.71 ± 0.02a
5 March 2018	H_2_CN_2_	64.50 ± 1.39ab	13.83 ± 1.89a	21.03 ± 1.76a	0.33 ± 0.03a	11.40 ± 0.56a	0.74 ± 0.01a
13 May 2018	Water	70.50 ± 2.11b	9.71 ± 1.84a	18.57 ± 1.25a	0.26 ± 0.02a	12.15 ± 0.64a	0.72 ± 0.01a
4 August 2018	Water	72.77 ± 2.50b	10.68 ± 2.38a	16.57 ± 2.38a	0.23 ± 0.03a	12.13 ± 1.47a	0.79 ± 0.03a

Buds were processed for analysis 24 h after treatment by submergence for 30 s in 1.25 % (w/v) hydrogen cyanamide or water.

Mean and standard deviation values followed by different letters indicate significant differences at *P* < 0.05 evaluated using one-way ANOVA followed by Tukey’s multiple comparison test (*n* = 3) independently for each year.

### Ultrastructure analysis of the grapevine bud shoot apical meristem

While flow cytometry provided quantitative insights into cell cycle state, it cannot reveal the subcellular changes accompanying dormancy transitions. Therefore, we used TEM to examine the ultrastructure of the shoot apical meristem, focusing particularly on organelle development and cellular organization. The TEM observations were conducted on shoot primordia from primary buds collected in March, May and August 2017. Semi-thin sections at 1 µm were made to trim resin-embedded buds until the area of the shoot primordium was visible (Fig. 1A). Morphology observations were made at two regions of the SAM, i.e. the central zone (CZ) and peripheral zone (PZ) (Fig. 1B).

**Fig. 1.**
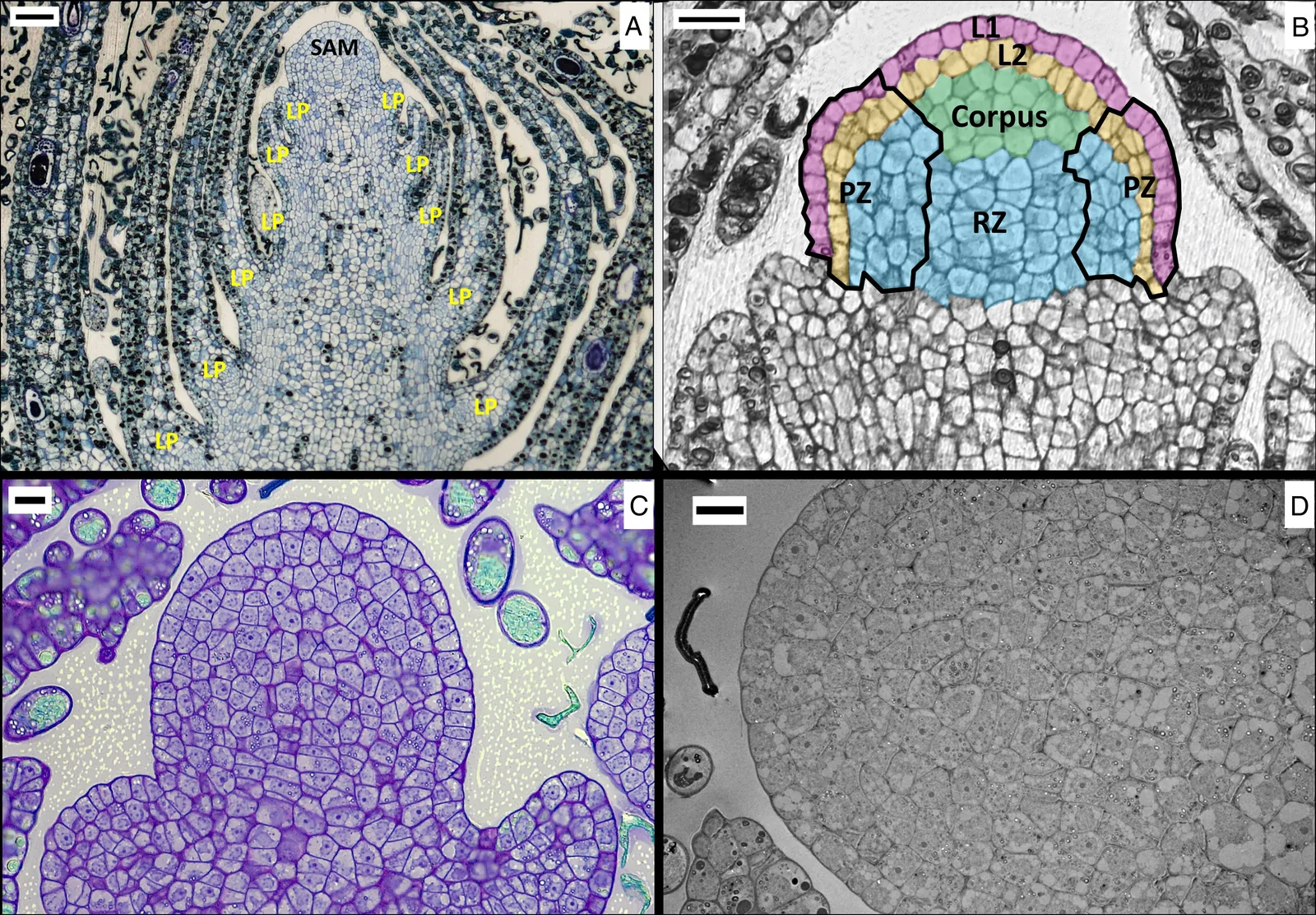
Median longitudinal section of the vegetative shoot of the *Vitis vinifera* cv. ‘Cabernet Sauvignon’ primary bud. (A) Light micrograph showing the zone of the shoot apical meristem (SAM) and six leaf primordia (LP)/nodes. Scale bar = 50 µm. (B) Schematic interpretation of the SAM zonation according to Mullins *et al.* (1992) showing the central zone consisting of a tunica (L1 and L2 cell layers) and corpus, peripheral zone (PZ) and rib meristem zone (RZ). Scale bar = 20 µm. (C) Representative light micrograph of the apical dome of the SAM and two leaf primordia. Scale bar = 10 µm. (D) Representative low-magnification electron micrograph of the apical dome. Scale bar = 10 µm.

Figures 2–5 show representative TEM images of the SAM of grapevine buds at each time or treatment condition in the tunica (Fig. 2), PZ (Fig. 3), rib zone (RZ, Fig. 4) and leaf primordia (LP, Fig. 5). Starch grains were present in cells throughout the SAM at each time or treatment condition, with relative abundance being higher in March and August than in May buds. The nucleolus and heterochromatin were the most apparent structures in the CZ area. Heterochromatin patches at the periphery of the nuclear envelope were clearly visible in the nucleus of buds collected in March and treated with cyanamide (Fig. 2A, B). A nucleolus can be identified as a densely stained spherical structure inside a nucleus. Meanwhile, euchromatin usually appeared as a thin fibre scattered throughout the nucleoplasm, which then began to condense as the cells prepared for cell division, forming a small densely stained patch of heterochromatin. The cell walls throughout the SAM of buds collected in March and August appeared thicker than those collected in May. Endoplasmic reticulum was visible in bud cells collected in May (black arrowheads, Fig. 2E, F) and relatively absent in buds collected in March (Fig. 2C, D) and August (Fig. 2G, H). Numerous osmiophilic organelles were visible (indicated by the asterisks), especially in buds collected in August (Fig. 2H); however, no detailed internal membrane structure, e.g. grana or cristae, could be observed, making it difficult to identify these organelles.

**Fig. 2.**
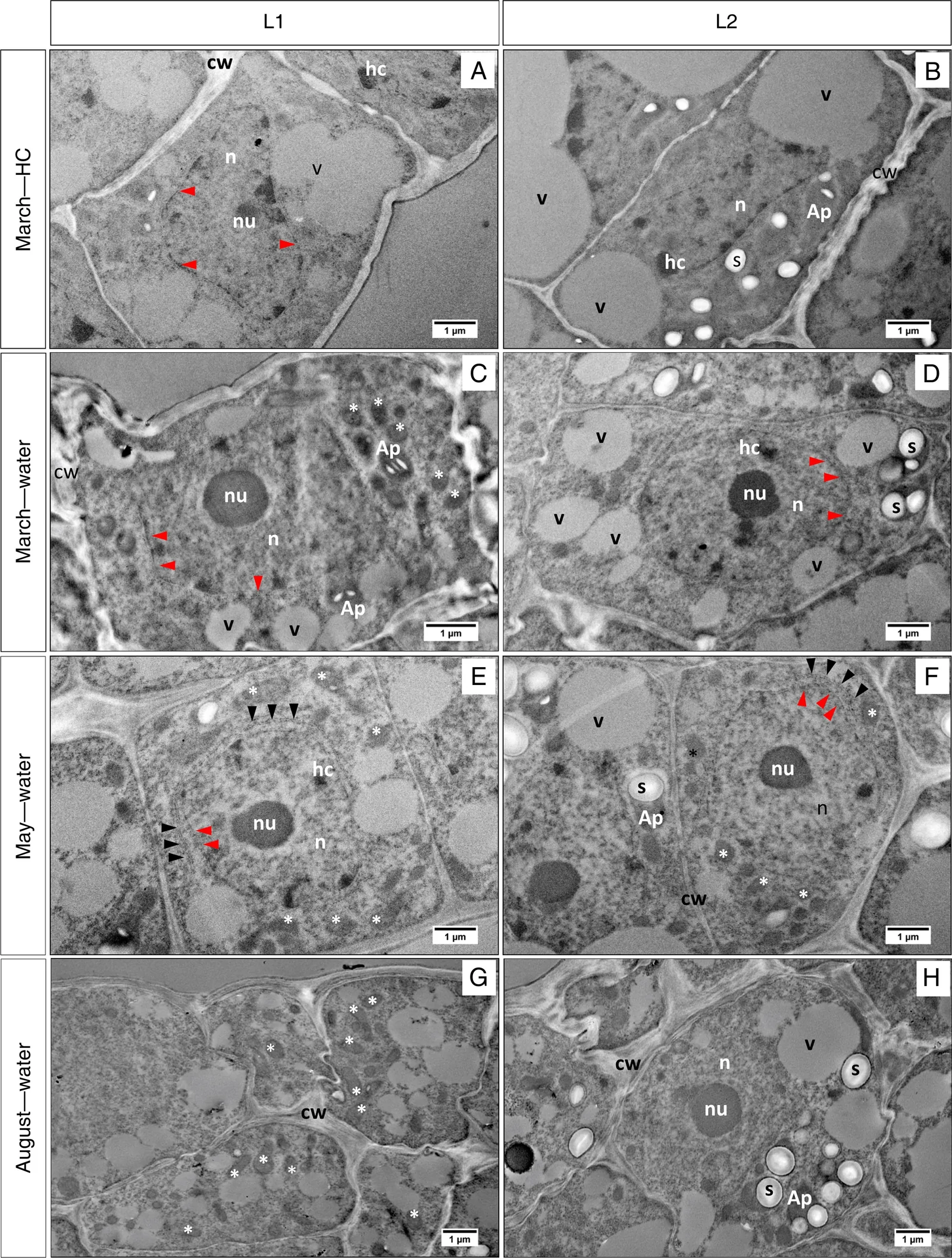
Electron micrographs at the tunica layers (L1 and L2) at the shoot apical meristem. (A, B) Tunica of buds collected in March treated with hydrogen cyanamide. Nuclei (n) with nucleoli (nu) and heterochromatin (hc) patches were highlighted at both L1 and L2. Starch grains (s) were visible at L2 and some located inside amyloplast (Ap). v, vacuole. (C, D) Tunica of buds collected in March treated with water. Large nucleoli (nu) were observed inside the nuclei (n) at L1 and L2. Also visible are a few heterochromatin (hc) patches, the double membrane of nucleus envelope (arrowheads) and numerous small vacuoles (v). Thick cell walls (cw) appeared at L1 of tunica. Asterisks indicate numerous osmiophilic organelles, presumably proplastid or lipid bodies. (E, F) Tunica of buds collected in May treated with water. Endoplasmic reticulum (black arrowheads) can be observed. Also, visible nuclei (n), large nucleoli (nu), double membrane of nucleus envelope (red arrowheads), heterochromatin (hc) patches and small vacuoles (v). Numerous osmiophilic organelles are also visible (asterisks). (G, H) Numerous osmiophilic organelles (asterisks) and thick cell walls are highlighted in the tunica layers of buds collected in August treated with water.

**Fig. 3.**
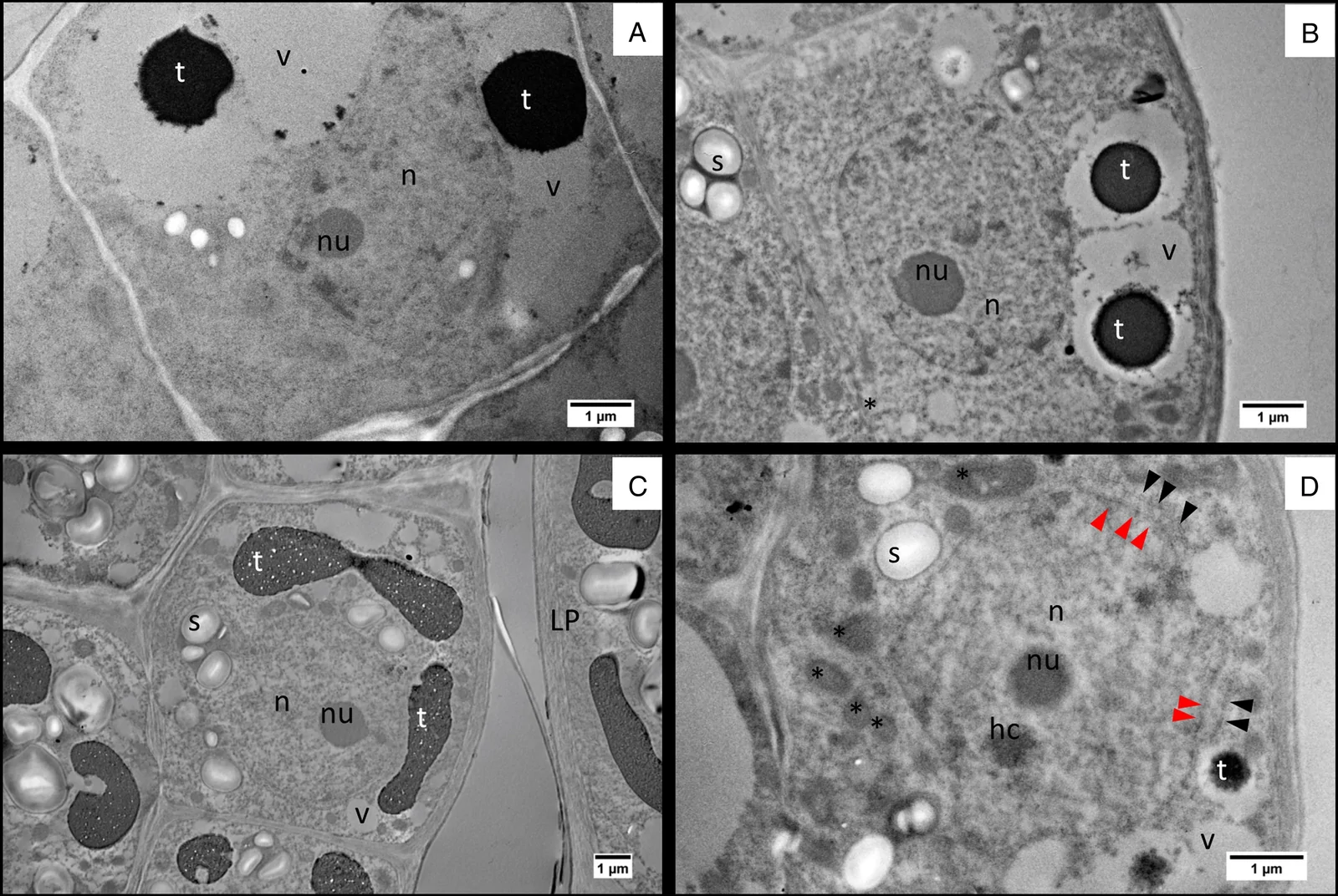
Transmission electron micrograph at the peripheral zone of the shoot apical meristem of grapevine buds collected in March (A, treated with hydrogen cyanamide; B, treated with water), May (C) and August (D). Tannin depositions (t) were found inside the vacuole of cells in the peripheral zone. Other abbreviations are defined in the legend of Fig. 2.

**Fig. 4.**
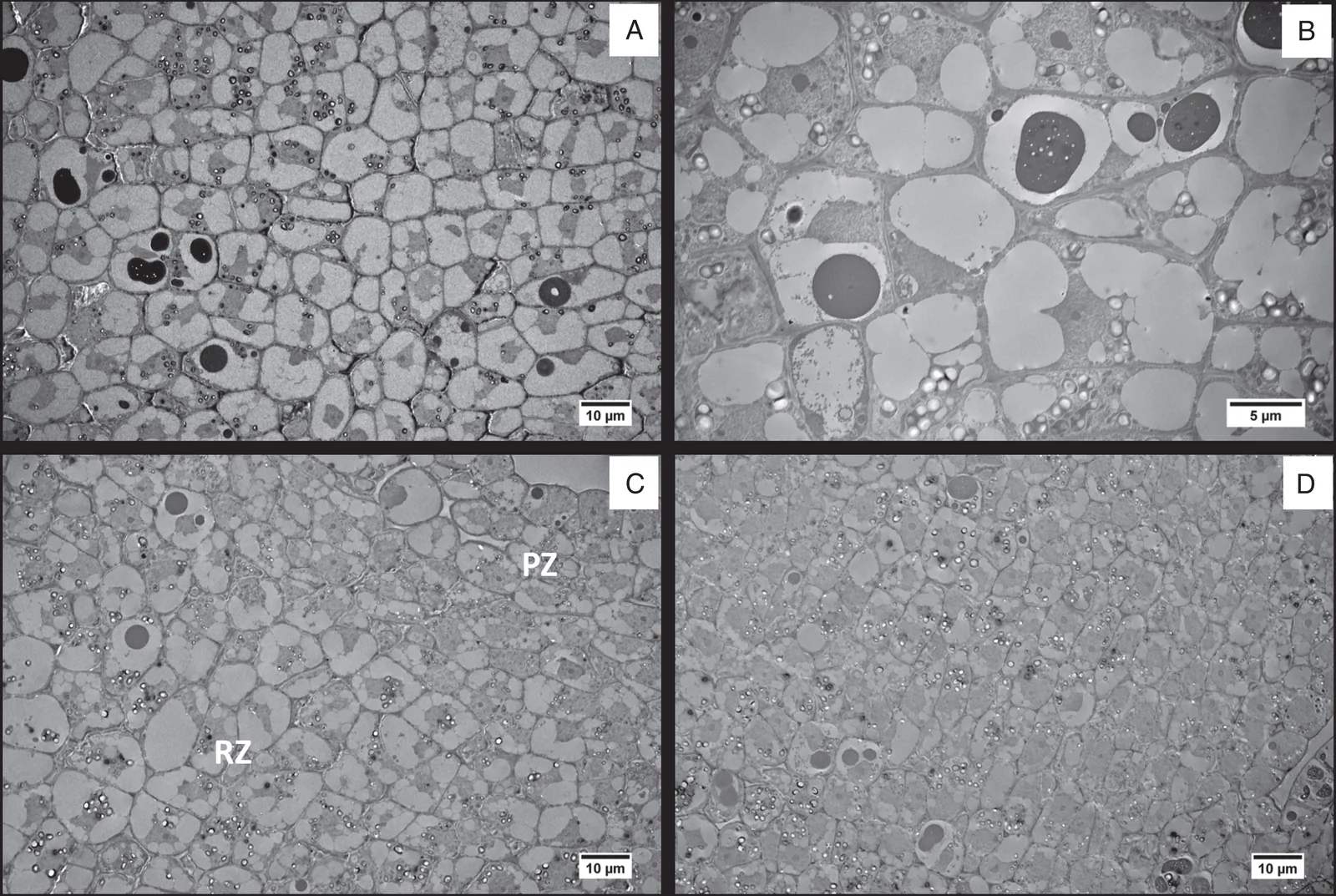
Transmission electron micrograph at the rib meristem zone (RZ) of the shoot apical meristem of grapevine buds collected in March (A, treated with hydrogen cyanamide; B, treated with water), May (C) and August (D). Cells were relatively larger at RZ compared with the peripheral zone (PZ) mainly due to increased vacuolation. Tannin deposition and starch grains can also be found in this zone.

**Fig. 5.**
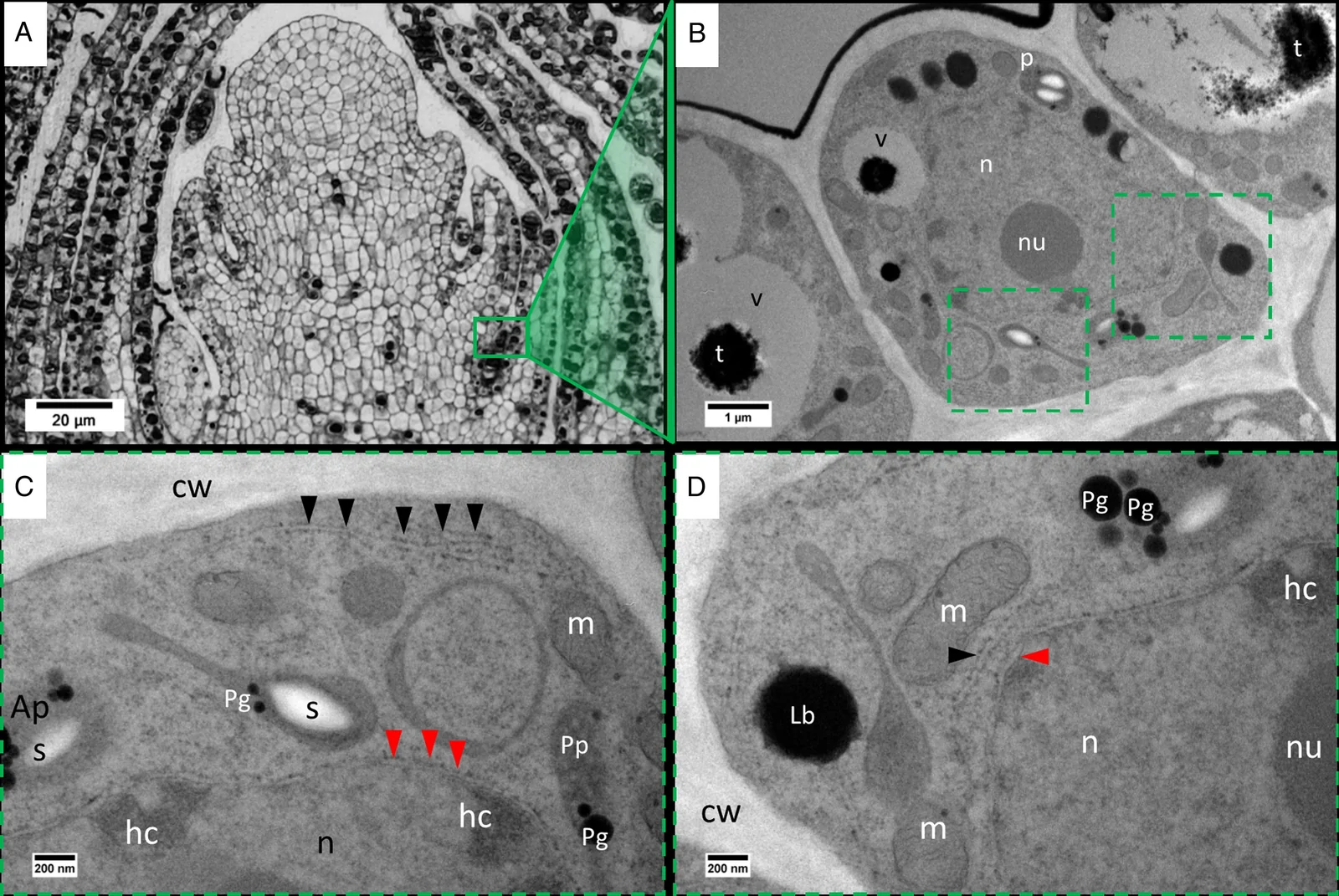
Micrographs of cells in the leaf primordium area of mature dormant buds (March, hydrogen cyanamide-treated) of *Vitis vinifera* cv. ‘Cabernet Sauvignon’ reveal cellular ultrastructure of several organelles. (A) Light micrograph of shoot apex showing the area of observation, i.e. the leaf primordia (green rectangle). (B) Representative TEM image of a cell in the leaf primordium area. Visible are nucleus (n), nucleolus (nu), amyloplast (Ap) and vacuole (v) with tannin deposition (t). (C, D) Higher magnification TEM images of two areas indicated by dashed rectangles in (B). Visible organelles are heterochromatin (hc), proplastid (Pp), amyloplast (Ap), plastoglobulin inside plastid (Pg), osmiophilic organelles, presumably lipid bodies (Lb), mitochondria (m), starch grain (s) and cell wall (cw). Double membrane nuclear envelope (red arrowheads) and endoplasmic reticulum (black arrowheads) are also visible.

Having characterized the cellular features of the tunica layers, we examined the peripheral and rib zones of the SAM. These zones showed distinct patterns of vacuolation and metabolite accumulation. Cells at the peripheral zone were identified by the presence of tannin deposition (Fig. 3). The vacuoles were found to be larger at the rib meristem zone and occupied a significant part of the cells (Fig. 4), in contrast to cells at the tunica, which were largely occupied by the nucleus (Fig. 2).

Finally, to understand how cellular organization differs between meristematic and differentiating tissues, we examined the ultrastructure of leaf primordia, which revealed notable differences in organelle development and metabolic status compared with the SAM proper. Ultrastructural details of the nucleus were clearest in the LP area (Fig. 5A), showing the double membrane of the nuclear envelope (red arrowheads), nucleolus and heterochromatin at the periphery of the nucleus (Fig. 5C). Also visible were the deposition of tannins inside the vacuole (Fig. 5B), starch grains inside a plastid (Fig. 5C), plastoglobulins inside a proplastid (Fig. 5C), endoplasmic reticulum (Fig. 5C, D; black arrowheads) and the cristal membrane in the mitochondria (Fig. 5D).

## DISCUSSION

This study reveals fundamental insights into the cellular basis of bud dormancy in grapevine, challenging several longstanding assumptions about dormancy regulation in woody perennials. Our key finding is that cell cycle arrest persists even as dormancy depth declines substantially. This challenges the traditional model linking dormancy release to cell cycle activation. Combined with our detailed ultrastructural analysis of the shoot apical meristem, these observations indicate that dormancy transitions involve structural changes in cellular organization and molecular reprogramming. The cell cycle appears to be subordinate to, rather than a driver of, dormancy transitions.

This study represents the first investigation of woody perennial buds combining cell cycle analysis via FCM with detailed electron microscopy across multiple time points, achieving unprecedented temporal breadth, developmental precision and spatial resolution. The FCM analysis was conducted on bulk tissue, despite careful dissection to isolate the primary bud from secondary and tertiary buds, bracts, fibres and scales. This limits the spatial resolution of our conclusions. In particular, the SAM comprises functionally distinct zones (the central zone maintaining pluripotent stem cells, and the peripheral and rib meristem zones where organogenesis and early differentiation occur) that may differ in their cell cycle dynamics during dormancy transitions. If early cell cycle reactivation were confined to a small number of cells within a specific micro-region, this signal could be diluted below the detection threshold by the dominant G1-arrested population of the bulk tissue. However, our TEM observations surveyed cells across all SAM zones (tunica, peripheral zone, rib meristem and leaf primordia) and did not detect mitotic cells or condensed chromosomes, indicative of active division, at any time point. In addition, the consistency of the cell cycle profiles across two independent seasons and the concordance with Signorelli *et al.* (2026) support the conclusion that G1 arrest is pervasive rather than an artefact of signal dilution. Nevertheless, future studies employing laser-capture microdissection or single-cell transcriptomics would be valuable for resolving whether zone-specific cell cycle dynamics differ during the dormancy-to-quiescence transition. Furthermore, the co-chopping method provides internal validation that our FCM analysis is robust.

A methodological consideration in our FCM analysis is the relatively high debris factor (59–79 %) in the nuclear suspensions, which is characteristic of woody perennial tissues rich in tannins, phenolics, and other secondary metabolites that generate particulate debris during chopping-based nuclear extraction (Loureiro *et al.*, 2007; Hermawaty *et al.*, 2022). The Watson pragmatic algorithm accommodates such background by modelling debris as a component distinct from the cell cycle peaks, fitting G1 and G2 distributions on the basis of peak shape rather than requiring a clean baseline (Watson *et al.*, 1987). The CV values in our preparations (10–14 %) fall within acceptable ranges for plant FCM (Galbraith *et al.*, 2021), indicating adequate peak resolution for reliable phase estimation. Moreover, the consistency of cell cycle profiles across two independent seasons, together with complementary TEM observations showing no mitotic figures in the SAM, supports the reliability of our FCM data. Representative histograms illustrating the gating strategy and peak resolution are provided in Supplementary Data Fig. S3.These findings align with recent work at the same Margaret River site (Velappan *et al.*, 2022; Signorelli *et al.*, 2026). The two studies assessed different genotypes, cv. ‘Merlot’ and cv. ‘Cabernet Sauvignon’, showing a congruent and pronounced but transient peak in dormancy depth during late summer (BB_50_ ∼300 d in February), followed by a biphasic decline through autumn and winter. Critically, this decline occurred despite negligible chilling accumulation in early autumn, reinforcing that the physiology of dormancy in Mediterranean climates is poorly explained by chilling models. Signorelli *et al.* (2026) further demonstrated that such a transient peak in dormancy was coupled with reduced respiration, decreased water content and apoplastic isolation. The consistency of these patterns across cultivars and years at this site provides strong evidence that the dormancy regulation mechanisms we observe represent robust physiological responses rather than cultivar- or season-specific anomalies. Importantly, these dynamics call into question the appropriateness of the widely used delineations of bud dormancy into para-, endo- and ecodormant states. By these definitions, the buds would be considered ecodormant from early autumn, well before chilling exposure, which has been held as the trigger to establish ecodormancy.

### Bud cell cycle progression was halted at G1 phase

Arrest at G1 persists throughout the dormancy cycle despite dramatic changes in dormancy depth, as seen in this study and Signorelli *et al.* (2026). While previous studies have shown G1 arrest during deep dormancy (Pawłowski *et al.*, 2004; Velappan *et al.*, 2017), the persistence of a relatively high fraction of G1 despite considerable reductions in depth of dormancy raises important questions about the relationship between cell cycle progression and bud burst competence. Whether these patterns are unique to grapevine or represent general features of woody perennial dormancy remains to be determined through application of this experimental framework to other species.

The persistence of G1 arrest in grapevine buds differs notably from seed dormancy, where dormancy enforces metabolic quiescence and embryos often display a predominance of 2C nuclei. In seed, water content can fall below 10 %, a condition in which chromatin is highly condensed while seed rehydration decondenses the chromatin and restarts metabolic activity (El-Maarouf-Bouteau, 2022). During imbibition by non-dormant seeds, cell cycle resumption is frequently detected as a rising fraction of 4C nuclei (and increasing 4C/2C ratio) in embryonic tissues. In *Brachypodium distachyon* embryos, FCM showed a progressive increase in 4C nuclei (with a concomitant decline in 2C nuclei) across the first ∼20 h after imbibition, with the 4C/2C ratio increasing accordingly (Wolny *et al.*, 2018). In wild oat (*Avena fatua*) caryopses, dormancy release was associated with cell cycle activation before coleorhiza emergence, whereas dormant and ABA-treated caryopses displayed inhibited or delayed cell cycle activation (Kępczyński *et al.*, 2021). Together, these seed studies support the view that nuclear DNA content (or 4C/2C ratio) dynamics can be a reliable indicator of germination competence during dormancy release in desiccation-tolerant organs. In contrast, grapevine buds maintain ∼40–45 % water content throughout dormancy, a condition that is sufficient to support metabolic activity. Signorelli *et al.* (2026) demonstrated that peak dormancy at the Margaret River site coincided with minimal hexoses (glucose, fructose, trehalose, sucrose), reduced respiration and decreased S-phase cells despite this maintained hydration. Strong correlations between dormancy depth and metabolite levels (fructose, *r* = −0.53; malate, *r* = −0.52; citrate, *r* = −0.50) indicate that G1 arrest in grapevine buds reflects coordinated metabolic suppression rather than desiccation-induced constraint. These observations suggest that while G1 arrest may be conserved across plant organs, the mechanisms regulating cell cycle reactivation differ fundamentally: desiccation and rehydration in seeds versus metabolic regulation in buds.

The mitotic index in this study did not reflect dormancy depth. The majority of buds had 2C nuclear DNA content (G1 phase), with the proportion of G1 cells increasing towards dormancy release in May and August. This accumulation of G1-arrested cells likely reflects slow cell cycle progression rather than complete arrest, given that buds maintain ∼40 % H_2_O compared with ∼10 % in seeds. It is worth noting that FCM measures cell cycle phase distribution but not kinetics and that seed germination occurs rapidly (nuclear changes within 24 h), whereas bud break required 3 weeks after single-node planting in August, even with cyanamide treatment. Vergara *et al.* (2016) demonstrated moderate increases in *VviCDKA*, *VviCDKB2* and *VviCYCA1* expression within 48 h of cyanamide treatment, but not *VviCYCB* or *VviCYCD3.2*, indicating that cyanamide does not immediately activate the progression to the mitotic phase. This activation is consistent with the temporal framework described by Meitha *et al.* (2018) using chilled quiescent ‘Crimson Seedless’ single-node explants grown at 23 °C, suggesting that stimulus responses and re-establishment of meristem identity precede mitosis, with DNA-replication licensing transcripts elevated within 3 h and increases in A- and B-type cyclins/CDKs becoming evident by ∼24 h, while visible bud burst (EL4) occurs only days later (50 % bud burst in 7–10 d). Signorelli *et al.* (2026) observed that by September, when natural bud burst was imminent, cells in G2 phase increased alongside sugar–phosphate accumulation (Fru6P, Glc6P, Suc6P), suggesting that cell division reactivation occurs very late in the dormancy release process and may be triggered by sugar signalling pathways.

Molecular regulation during dormancy is beyond the scope of this study, but recent work helps contextualize our cytological evidence for prolonged G1 dominance and late reactivation. Multi-omics analysis of cyanamide-treated grapevine buds showed that dormancy release is accompanied by early shifts in energy metabolism and hormone signalling, along with upregulation of cytoskeleton components, including α-tubulin, consistent with cytoskeletal remodelling as part of a priming phase that can precede mitotic entry (Khalil-Ur-Rehman *et al.*, 2019). In seeds, dormancy–germination control is mediated by ABA-GA networks centred on ABI5 and DELLA/RGL2 modules, illustrating how signals for cell cycle arrest and progression can be imposed even before a visible proliferation event takes place (Farooq *et al.*, 2022; Zhao *et al.*, 2022; Sajeev *et al.*, 2024). This finding aligns with our interpretation that grapevine buds maintain hydration while remaining metabolically constrained until very late in the release process.

### Subcellular morphology showed the characteristic of cells undergoing dormancy

In perennial trees, the regulation controlling establishment and release of bud dormancy is less well understood than the regulation of growth cessation and bud set (Singh *et al.*, 2017). Evaluation of dormancy depth still largely relies on bud burst experiments under forcing conditions. This approach has limitations in standardization of procedure and data analysis (Dennis, 2003; Alvarez *et al.*, 2018). Here we used FCM to measure the mitotic index and TEM to observe morphology as alternative approaches to objectively defining dormancy stages in grapevine buds.

The cellular ultrastructure of the SAM region showed several features in common with the SAM from other plant species at a dormant stage. Cells at the central and peripheral zone were similar to those observed in *Salix* sp. (Berggren, 1985). These cells had nuclei occupying a significant part of the cell and undeveloped organelles such as proplastids, and were relatively small with dense cytoplasm. In contrast, cells at the rib zone are easily identified by the less dense cytoplasm due to increasing vacuolation in the cells and deposition of tannin inside the vacuole (Berggren, 1984).

Microscopic observation in the *Helianthus annuus* shoot apex showed the presence of starch grains in quiescent central zone cells but they were relatively absent from more active peripheral cells (Sawhney *et al.*, 1981). In the grapevine buds observed in this study, starch grains were present in both central and peripheral zones at all time points, suggesting that these cells were mitotically less active at the time of sampling. This contrasts with Norway spruce, where the absence of starch (together with callose at plasmodesmata) was identified as an ultrastructural indicator of endodormancy, while the shift towards ecodormancy involved tissue-specific starch hyperaccumulation and subsequent degradation alongside dynamic changes in plastids and mitochondria (Guzicka *et al.*, 2018). In *Prunus cerasus*, starch development was observed at the SAM following chilling of quiescent flower buds, whereas quiescent buds without chilling developed more abundant mitochondria and lacked starch grains (Felker *et al.*, 1983). We observed a comparable pattern, with fewer starch grains in May buds (quiescent) than August buds (quiescent and chill-accumulated), supporting the view that starch abundance can reflect shifts in organelle activity and reserve mobilization during late dormancy progression rather than a simple dormancy stage marker.

As mentioned earlier, our data showed that starch grains also appeared relatively abundant in grapevine buds collected in March, when the depth of dormancy was highest and chilling was still absent. This observation gains significant context from climate data at the same site (Velappan *et al.*, 2022). Between January and May, dormancy depth declined substantially (see next section). However, cumulative chilling remained negligible during this period. This indicates that in Mediterranean climates like Margaret River, the substantial decline in dormancy depth can occur independently of meaningful chilling accumulation. The presence of starch grains in grapevine buds likely indicates two distinct physiological states. First, starch marks the dormant state during summer–autumn, when dormancy is of the greatest depth but chilling is absent. Second, starch accumulates later in association with chilling exposure in winter.

Metabolomic data from Signorelli *et al.* (2026) support this interpretation. The peak dormancy period (January–February) is characterized by high levels of starch precursors (ADP-glucose). In contrast, the later winter period (August) shows sugar mobilization (see next section). During the early dormancy phase (January), buds transition from quiescence to dormancy. High levels of myo-inositol, shikimate and tartrate were observed during this period. This suggests investment in protective compounds and preparation for dormancy. The accumulation of raffinose family oligosaccharides (RFOs) peaked in autumn (May), coinciding with the onset of cooler temperatures and shorter photoperiods, consistent with their roles in cold protection and osmotic adjustment. By late winter (August), chilling had accumulated substantially. There was a marked increase in metabolic activity during this period. Elevated levels of glucose, fructose, trehalose and sucrose, as well as sugar–phosphates, indicate preparation for growth resumption. Further, the disappearance of starch grains, which coincided with the development of mitochondria, could only be observed in the more differentiated cells of leaf primordia (Fig. 5).

Numerous osmiophilic organelles were observed in the SAM, particularly in August buds. These are tentatively labelled as putative lipid bodies in the micrographs (Fig. 5) on the basis of their size, electron density and morphological similarity to lipid bodies described in other woody species, but their identity could not be confirmed, as internal membrane structures were not resolvable at the magnifications used. Confirmatory approaches such as lipid-specific fluorescent staining (e.g. Nile red) or immunolabelling would be required for definitive identification. In other woody species, similar structures have been identified as lipid bodies that serve both metabolic and regulatory functions. In the evergreen angiosperm *Rhododendron maximum*, osmiophilic lipid bodies located at cell peripheries serve as energy reserves (Lynch and Rivera, 1981). In *Betula pubescens*, lipid bodies deliver 1,3-β-glucanase to plasmodesmata, facilitating the removal of callose plugs that restrict cell-to-cell communication during dormancy (van der Schoot *et al.*, 2011; Paul *et al.*, 2014). We observed changes in cell wall thickness between sampling dates. However, we did not detect clear evidence of plasmodesmatal sphincters like those described in *B. pubescens*. In that species, sphincter structures containing 1,3-β-D-glucan block plasmodesmata during endodormancy and disappear upon dormancy release (Rinne *et al.*, 2001; Rinne and van der Schoot, 2004). The importance of such structures in dormancy regulation is highlighted by studies in transgenic hybrid aspen (*Populus tremula* × *Populus tremuloides*), where the absence of plasmodesmata sphincters prevents proper dormancy development in response to short photoperiod (Tylewicz *et al.*, 2018).

As noted above, apoplastic isolation occurs during peak dormancy. Signorelli *et al.* (2026) demonstrated that this isolation, as measured by reduced uptake of apoplastic dyes, may serve to metabolically insulate the bud during the period of deepest dormancy. This prevents resource drain to the cane while maintaining internal metabolic homeostasis. Our findings suggest that if similar regulatory mechanisms exist in grapevine, they may involve different cellular structures or molecular pathways than those described in other woody perennials. The changes in cell wall thickness at the SAM, as well as the presence of starch grains and lipid bodies, were also reported in the SAM of *Cunninghamia lanceolata* during the transition from dormancy to active growth (Xu *et al.*, 2016). In this study, differences in cell wall thickness were more pronounced in the tunica. Buds collected in March and August had thicker cell walls than those collected in May. Apart from cell wall thickening, we did not find evidence of plasmodesmata sphincters and lipid bodies in our study.

### Integration of dormancy physiology with seasonal climate patterns

The cellular observations in this study must be contextualized within the seasonal climate patterns characteristic of Mediterranean grape-growing regions. At the Margaret River site (33–34°S) three studies were conducted (Velappan *et al.*, 2022; Signorelli *et al.*, 2026; this study). Winter chilling accumulation begins only in late autumn (May onwards), reaching modest levels by late winter (∼400–600 DPCU; Supplementary Data Fig. S2). The climate is characterized by a Mediterranean pattern: summers are hot and dry (mean temperatures 15–26 °C), autumns are mild and winters are cool and wet (mean temperatures 8–18 °C). Spring warming triggers natural bud burst in early September (Velappan *et al.*, 2022).

Critically, the substantial decline in dormancy depth observed between late summer and mid-autumn (from BB_50_ ∼300 d in February to ∼60 d in May) occurs during a period of negligible chilling exposure (Supplementary Data Fig. S2). This temporal disconnect between dormancy release and chilling accumulation challenges traditional chilling-centric models. It suggests that photoperiod, temperature fluctuations and endogenous developmental programmes play dominant roles in the initial phases of dormancy release in this cultivar and climate. The photoperiod at this latitude decreases from ∼14 h at the summer solstice (22 December) to 10 h by the winter solstice (21 June). This provides a strong environmental cue for seasonal transitions that precedes substantial chilling accumulation.

The metabolic data of Signorelli *et al.* (2026) reveal characteristics of this early phase of dormancy release. Respiration rates decline (from ∼4 nmol O_2_ min^−1^ g^−1^ FW in February to ∼2 nmol O_2_ min^−1^ g^−1^ FW in May). Water content is reduced (from ∼0.65 to ∼0.45 g H_2_O g^−1^ FW). Raffinose accumulates. Metabolic reorganization occurs. Notably, these changes differ from the metabolic reactivation typically associated with chilling responses. The respiratory suppression during this period is particularly striking because it occurs despite declining dormancy depth. This suggests that dormancy release involves removal of growth-inhibitory signals rather than activation of growth-promoting processes. Only later, in late winter (August), when substantial chilling has accumulated, does respiration increase again (to ∼3.5 nmol O_2_ min^−1^ g^−1^ FW). This occurs coincident with sugar mobilization, increased sugar-phosphate levels and the appearance of cells in G2 phase.

These findings collectively indicate that dormancy in grapevine operates through multiple, partially independent regulatory modules. The initial establishment of deep dormancy in summer appears to involve active metabolic suppression, apoplastic isolation and coordinated reduction in cell cycle progression and respiratory metabolism. The first phase of dormancy release in autumn occurs through removal of these inhibitory mechanisms and appears to be driven primarily by photoperiod cues and temperature fluctuations rather than chilling accumulation per se. Winter chilling may serve a permissive or modulating role. It potentially contributes to the second, slower phase of dormancy decline observed between May and August. It may also facilitate the metabolic reactivation and cell cycle re-entry that prepares buds for burst. However, our data clearly show that substantial dormancy release can occur in the absence of significant chilling, challenging the view that winter chilling is the primary driver of dormancy release in grapevine.

### Conclusions

Our integrated analysis reveals fundamental new insights into bud dormancy regulation in grapevine. Four key findings challenge current understanding. First, substantial dormancy release (5-fold decrease in BB_50_) occurs independently of winter chilling, indicating that photoperiod and temperature fluctuations, rather than chilling, drive the initial phase of dormancy release in Mediterranean climates. Second, the vast majority of cells remain arrested in G1 phase regardless of dormancy status, with cell cycle re-entry occurring only very late, suggesting that bud burst capacity is determined by removal of constraints on growth rather than cell cycle reactivation. Third, starch accumulation occurs both in deeply dormant pre-winter buds and in chill-exposed winter buds, indicating multiple roles as markers of metabolic quiescence and preparation for growth resumption. Fourth, by coherence with two independent datasets, dormancy transitions involve coordinated changes in metabolism, respiration, water relations and apoplastic connectivity, with dormancy establishment characterized by active suppression followed by sequential release of these mechanisms.

This study represents the first investigation of its kind in woody perennials. Whether the patterns we observe represent unique features of grapevine biology or general characteristics of woody perennial dormancy regulation remains to be determined. The coordination of metabolic suppression, apoplastic isolation and cell cycle arrest suggests a multi-layered regulatory system integrating endogenous developmental programmes with environmental signals. Application of this experimental framework to other species and climates will determine which aspects of dormancy regulation are conserved versus species-specific adaptations.

## Supplementary Material

mcag218_Supplementary_Data

## SUPPLEMENTARY DATA

Supplementary data are available at *Annals of Botany* online and consist of the following. Table S1: hourly temperature data retrieved from the weather station adjacent to the site, together with calculated chilling data from March to August, inclusive, for 2017–18. Figure S1: view of mature compound grapevine buds. Figure S2: accumulation of chilling hours for the two years of study. Figure S3: flow cytometry histogram for cell cycle analysis. File S1: sample preparation and processing for general morphology and ultrastructure of grapevine bud using light and transmission electron microscopy.
