# Supplementary material for: Growth competence develops independently of cell division: uncoupling dormancy and cell cycle in grapevine buds
Source: Ann Bot. 2026 Jul 18;138(5):1976–89. doi: 10.1093/aob/mcag218 (PMC13715000; doi:10.1093/aob/mcag218)
Supplement: Supplementary file 1 [file mcag218_supplementary_data.docx]

**Table S1.** The analysis of flow cytometry data on the population of nuclei stained with Propidium Iodide. This analysis was conducted using a gating strategy in accordance with the established protocol by Hermawaty et al. (2022). This methodology facilitated the precise enumeration of nuclei populations, enabling the calculation of the proportion of nuclei in each phase of the cell cycle, specifically G1, S, and G2.

| Year | Month | Replicate | Treatment | Total events | PI positive | PI nuclei | Intact nuclei | G1 (%) | G2 (%) | S (%) | CV |
| --- | --- | --- | --- | --- | --- | --- | --- | --- | --- | --- | --- |
| 2017 | March | 1 | Water | 196665 | 21366 | 10607 | 7730 | 50.8 | 40.3 | 8.23 | 14.6 |
|  | March | 2 | Water | 165045 | 19518 | 9730 | 6884 | 40.4 | 39.6 | 16.3 | 12.3 |
|  | March | 3 | Water | 164745 | 17127 | 8202 | 6015 | 38.7 | 38.1 | 18.4 | 11.7 |
|  | March | 1 | Cyanamide | 134415 | 15398 | 7657 | 6184 | 44.6 | 35.9 | 15.5 | 12.5 |
|  | March | 2 | Cyanamide | 127470 | 15771 | 8323 | 6871 | 36.7 | 32 | 25.2 | 9.96 |
|  | March | 3 | Cyanamide | 135045 | 16376 | 7989 | 6226 | 34.6 | 27.5 | 29.7 | 8.29 |
|  | May | 1 | Water | 295506 | 22736 | 11339 | 8137 | 58.6 | 28.1 | 13.3 | 13 |
|  | May | 2 | Water | 198435 | 18770 | 9999 | 7385 | 56.3 | 30.7 | 13 | 13.3 |
|  | May | 3 | Water | 112260 | 11386 | 6052 | 4650 | 60.3 | 29.8 | 9.91 | 13.8 |
|  | May | 4 | Water | 287387 | 25565 | 14006 | 10098 | 70.6 | 29 | 0.47 | 17.1 |
|  | May | 5 | Water | 305535 | 23571 | 12573 | 9636 | 62.9 | 27.8 | 9.31 | 14 |
|  | August | 1 | Water | 161220 | 17025 | 8241 | 5931 | 67.6 | 22.8 | 9.53 | 14 |
|  | August | 2 | Water | 160785 | 14356 | 7162 | 5318 | 61.3 | 19.8 | 18.3 | 10.7 |
|  | August | 3 | Water | 161040 | 17199 | 8977 | 6771 | 66.9 | 18.3 | 14.8 | 11.1 |
| 2018 | March | 1 | Water | 501640 | 12200 | 4896 | 3777 | 59 | 28.3 | 12.4 | 11.8 |
|  | March | 2 | Water | 401720 | 13204 | 5124 | 3887 | 62.3 | 20.3 | 16.3 | 10.9 |
|  | March | 3 | Water | 480166 | 13853 | 5228 | 3792 | 65.8 | 18.4 | 15.1 | 10.6 |
|  | March | 1 | Cyanamide | 586634 | 15824 | 6372 | 4357 | 63.6 | 19.6 | 16 | 10.8 |
|  | March | 2 | Cyanamide | 647560 | 18992 | 7371 | 4908 | 63.8 | 23 | 12.5 | 11.9 |
|  | March | 3 | Cyanamide | 661200 | 18993 | 7293 | 4701 | 66.1 | 20.5 | 13 | 11.5 |
|  | May | 1 | Water | 1337680 | 35231 | 14116 | 9516 | 69.5 | 19.7 | 9.67 | 12.4 |
|  | May | 2 | Water | 932520 | 35542 | 14802 | 9591 | 70.8 | 19.6 | 8.71 | 12.4 |
|  | May | 3 | Water | 815104 | 27456 | 10868 | 8106 | 68.4 | 17.6 | 12.3 | 11.2 |
|  | May | 4 | Water | 715440 | 25111 | 9555 | 7277 | 73.3 | 17.4 | 8.15 | 12.6 |
|  | August | 1 | Water | 1420506 | 54244 | 22549 | 9313 | 69.8 | 8.28 | 21.7 | 11 |
|  | August | 2 | Water | 1088491 | 35858 | 15568 | 7371 | 69.1 | 7.69 | 22 | 11.6 |
|  | August | 3 | Water | 992800 | 31717 | 13933 | 7418 | 71.1 | 9.22 | 18.4 | 13.8 |

**Figure S1.** View of mature compound grapevine buds. The compound consists with one primary latent bud which will produce the next season’s shoot (N+2), and two secondary latent buds (N+3_1,2_) which only grow when the primary bud die. Two cutting planes relative to the canes are shown above. (**1**) Longitudinal section in which cutting were made crossing all three buds (**2**) Transversal section (relative to cane) in which cutting made parallel to the two secondary buds. The median longitudinal view of the shoot apical meristem as shown in **Figure 1A** is achieved by making transversal cutting relative to the cane.

**Figure S2.**Accumulation of chilling hours for the two years of study. Arrows indicate the times of sampling in respective years.

**Figure S3**. Flow Cytometry Histogram for Cell Cycle Analysis. The proportions of cells in the G1, S, and G2 phases were determined by creating gates on an Event Count vs. PI FL2 histogram. The image above illustrates the gating positions for each cell cycle phase. The Event Count corresponds to the count of PI-stained nuclei, while the PI FL2 value represents the fluorescence intensity of the PI. This image was generated using the web-based flow cytometry analysis tool, Floreada (<https://floreada.io/>). Months are abbreviated Mar (March), May (May), Aug (August) and treatments abbreviated W (water) and H (cyanamide).

**Supplementary File 1**

Sample preparation and processing for general morphology and ultrastructure of grapevine bud using light and transmission electron microscope.

Materials

1. Buds of grapevine
2. Scalpel handle and blade (small #11 blade is preferable, but can use #24 blade as well)
3. Wooden handle, needle tip
4. Stereo microscope
5. Safe-Lock microcentrifuge tube 2 mL tube (Eppendorf® #0030120094)
6. PBS tablets pH 7.4 (Gibco® #18912014)
7. Glutaraldehyde 25% (Electron Microscopy Science #16220)
8. Formaldehyde 16 % (Electron Microscopy Science #15710)
9. Ultra-Low Viscosity Resin (Polyscience, Inc. #17706-1)
10. Flat bottom embedding capsule (Electron Microscopy Science #70021)
11. Microwave vacuum (PELCO BioWave®)

**Method**

Fixative solution:

1. Make 1X PBS pH 7.4 by adding dissolving 1 PBS tablets in 100 mL miliQ water.
2. Dilute 10 mL each of 25% Glutaraldehyde and 16% Formaldehyde stock solution in 80 mL 1X PBS (pH 7.4) from step 1. Final concentration is 2.5% glutaraldehyde and 1.6% formaldehyde in 1X PBS (pH 7.4)

*Note: This fixative solution can be stored up to 6 month in 4^0^C. Always bring fixative solution to room temperature before use. Glutaraldehyde works best at room temperature.*

Sample preparation (primary meristem dissection):

3. Make a parallel cut on both side the buds, parallel to secondary and tertiary buds (see Figure S1).

*Note: this will greatly aid complete fixation and also useful as a cutting place when putting sample in the embedding capsule.*

4. Into 1 mL fixative solution in 2 mL tube, put 2 dissected buds. More buds must be put in a bigger tube with more fixative solution, i.e 5 buds in 4 mL fixative. Invert the tube to wet the buds with fixative. At this point all bud will still be floating on the fixative solution.
5. Place tube (lid OPEN) inside the vacuum chamber and apply vacuum in cycle of 5 min vacuum/ release/ mix. Repeat vacuum cycle for at least 3 times and 5 times maximum.

Note: vacuum pressure use is ±20 inchHg.

6. Most of the buds should submerge to bottom of the tube and some may still float on the fixative solution at this point. Leave buds at room temperature without vacuum for overnight to continue the infiltration process.

*Notes:*

- *Buds that are successfully* ***submerged after vacuum cycle is the best sample*** *for further processing, nevertheless, buds that submerged after overnight infiltration will also give good results as well.*
- *It is* ***not preferable*** *to use buds that is not submerged after O/N infiltration although the buds might end up submerged after additional vacuum and infiltration time.*

7. Store buds in fixative solution at 4^0^C until further use.
8. For electron microscopy analysis, bud must be dissected into smaller size (at least 1 mm thickness and not more than 2 mm) by remove the scales carefully until about 3-4 layers left. Buds must always be covered with fixative solution during dissection.
9. Continue with tissue processing and embedding following Clode (2015) protocol for light and electron microscopy (see Table S2).

**Reference**

Ruzin, SE. 1999. *Plant Microtechnique and microscopy.* New York: Oxford University Press, pp.49 (with modification).

Li et al. 2014. A Robust chromatin immunoprecipitation protocol for studying transcription factor-DNA interactions and histone modification in wood-forming tissue. Nature protocol Vol. 9(9), pp. 2180-2193 (vacuum cycle).

**Table S2.** Procedure for processing and embedding grapevine bud for subsequent light and electron microscopy (Clode, 2015)

| Step | Solution | Procedure |
| --- | --- | --- |
| Rinse | PBS | 2 × 10 min |
| Post-fixation | 1% OsO_4_ in PBS | Microwaved 2 min ON/ 2 min OFF/ 2 min ON at 80 W with vacuum |
| Rinse | dH_2_O | 2 × 5 min |
| Dehydration | 30% ethanol | Microwaved 40 s at 240 W with vacuum and maintained under vacuum 1 min afterwards × 2 |
|  | 50% ethanol | Microwaved 40 s at 240 W with vacuum and maintained under vacuum 1 min afterwards × 2 |
|  | 70% ethanol | Microwaved 40 s at 240 W with vacuum and maintained under vacuum 1 min afterwards × 2 |
|  | 95% ethanol | Microwaved 40 s at 240 W with vacuum and maintained under vacuum 1 min afterwards × 2 |
|  | 100% ethanol | Microwaved 40 s at 240 W with vacuum and maintained under vacuum 1 min afterwards × 2 |
|  | Anhydrous ethanol | Microwaved 40 s at 240 W with vacuum and maintained under vacuum 1 min afterwards × 2 |
|  | Anhydrous acetone | Microwaved 40 s at 240 W with vacuum and maintained under vacuum 1 min afterwards × 2 |
| Infiltration | One part ULVR : five parts anhydrous acetone | Cycled under vacuum for 15 min in microwave (no watts) (4 min vacuum ON/ 1 min vacuum OFF) |
|  |  | Left on sample rotator for 24 h |
|  |  | Cycled under vacuum for 15 min in microwave (no watts) (4 min vacuum ON/ 1 min vacuum OFF) |
|  | One part ULVR : three parts anhydrous acetone | Cycled under vacuum for 15 min in microwave (no watts) (4 min vacuum ON/ 1 min vacuum OFF) |
|  |  | Left on sample rotator for 24 h |
|  |  | Cycled under vacuum for 15 min in microwave (no watts) (4 min vacuum ON/ 1 min vacuum OFF) |
|  | One part ULVR : one part anhydrous acetone | Cycled under vacuum for 15 min in microwave (no watts) (4 min vacuum ON/ 1 min vacuum OFF) |
|  |  | Left on sample rotator for 24 h |
|  |  | Cycled under vacuum for 15 min in microwave (no watts) (4 min vacuum ON/ 1 min vacuum OFF) |
|  | Three parts ULVR : one part anhydrous acetone | Cycled under vacuum for 15 min in microwave (no watts) (4 min vacuum ON/ 1 min vacuum OFF) |
|  |  | Left on sample rotator for 24 h |
|  |  | Cycled under vacuum for 15 min in microwave (no watts) (4 min vacuum ON/ 1 min vacuum OFF) |
|  | Five parts ULVR : one part anhydrous acetone | Cycled under vacuum for 15 min in microwave (no watts) (4 min vacuum ON/ 1 min vacuum OFF) |
|  |  | Left on sample rotator for 24 h |
|  | 100% ULVR | Maintained under vacuum for 24 h in vacuum oven |
|  |  | Placed in fresh resin, maintained under vacuum for 24 h in vacuum oven |
| Embedding | 100 % ULVR | Section mounted in flat-bottom BEEM capsule and maintained under vacuum for 1 h in vacuum oven |
|  |  | Vacuum for 1 h in vacuum oven |
|  | 100 % ULVR | Vacuum oven flooded with nitrogen gas × 5 cycles to produce oxygen-free environment |
|  |  | Cured under vacuum in oxygen-free environment at 65^0^C for 12 h |

PBS, phosphate-buffered saline; OsO_4_, osmium tetroxide (very toxic, to be used at CMCA facility only); dH_2_O, distilled water; ULVR, ultra-low viscosity resin
